# Population Kinetics and Mechanistic Aspects of *Saccharomyces cerevisiae* Growth in Relation to Selenium Sulfide Nanoparticle Synthesis

**DOI:** 10.3389/fmicb.2020.01019

**Published:** 2020-05-21

**Authors:** Farnoush Asghari-Paskiabi, Mohammad Imani, Sana Eybpoosh, Hashem Rafii-Tabar, Mehdi Razzaghi-Abyaneh

**Affiliations:** ^1^Department of Medical Physics and Biomedical Engineering, School of Medicine, Shahid Beheshti University of Medical Science, Tehran, Iran; ^2^Department of Mycology, Pasteur Institute of Iran, Tehran, Iran; ^3^Novel Drug Delivery Systems Department, Iran Polymer and Petrochemical Institute, Tehran, Iran; ^4^Department of Epidemiology and Biostatistics, Research Centre for Emerging and Reemerging Infectious Diseases, Pasteur Institute of Iran, Tehran, Iran

**Keywords:** *Saccharomyces cerevisiae*, selenium sulfide nanoparticles, sulfite reductase, modeling, Growth Kinetics

## Abstract

Biosynthesis of nanoparticles (NPs) by microorganisms is a cost- and energy-effective approach. However, how the production of NPs affects the population of producing organism remains as an unresolved question. The present study aimed to evaluate the kinetics of *Saccharomyces cerevisiae* growth in relation to synthesis of selenium sulfide nanoparticles by using a population model. To this end, the population of *S. cerevisiae* cells was investigated in terms of colony forming units (CFU) in the presence of the substrate in different time points. Fluctuation of sulfite reductase (SiR) activity, expression of *MET5* and *MET10* genes, and concentrations of sulfite and selenium were evaluated to support the population findings. CFU values in the test groups were lower than those in the control counterparts. The rise and fall of the SiR activity and *MET5* and *MET10* gene expression conformed to the variations of CFU values. The rate of reduction in the selenium and sulfite concentrations tended to decrease over the time. In conclusion, the cells population was negatively and positively affected by selenium and sulfite concentrations, respectively. The indirect relationship of the selenium ions concentration in the path analysis revealed that the product, selenium sulfide nanoparticles, caused this drop in *S. cerevisiae* cells population.

## Graphical Abstract

Purposed modeling scheme of the relationship between abiotic-gene expression variables and the population growth of *S. cerevisiae*. Solid arrows show the direct effects of independent variables on the dependent variables (*S. cerevisiae* population growth, gene expression, and sulfite reductase activity). Dashed arrows show the correlation coefficients among variables.

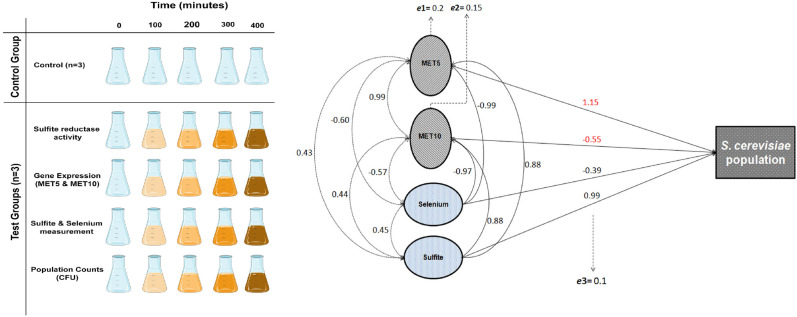

## Introduction

Green synthesis of nanoparticles (NPs), in comparison with conventional physicochemical approaches, is more simple and provides NPs with controlled size and morphology. Nowadays, great attention has been paid to develop NPs through biosynthesis approach by microorganisms, such as fungi, bacteria, plants, and plants extracts (Asghari et al., 2016).

Many studies have been carried out on bioconversion of selenium ions to NPs using selenium-based materials (Rajeshkumar et al., 2019). Forootanfar et al. (2014), assessed cytotoxicity and antioxidant activity of selenium NPs, which were biosynthesized by *Bacillus* sp. Msh-1, a bacterial strain isolated from sea. They found that selenium NPs had higher scavenging activity in comparison with selenium ions, while cytotoxic effect of biogenic selenium NPs on MCF-7 cell line was reduced considerably. Ahmad et al. (2015) biosynthesized stable selenium nanorods (Se Nrs) using *Streptomyces bikiensis*. The nanorods of selenium were able to induce death in Hep-G2 and MCF-7 cell lines.

In a study by Vogel et al. (2017), elemental selenium particles were produced by *Azospirillum brasilense*. They performed the experiment by two different substances. Interestingly, they found that selenate was non-toxic for *A. brasilense* and the bacterium did not reduce this oxyanion, while selenite, the other substrate, was toxic for *A. brasilense*. It was observed that the presence of selenite caused a long lag phase and just after initiation of cell growth, the bacterium converted selenite to NPs. *A. brasilense* was recognized to be highly sensitive to selenite, which was a burden on the selenium ion reduction path.

Earlier, we successfully biosynthesized selenium sulfide NPs using *Fusarium oxysporum* (Asghari-Paskiabi et al., 2018), and *Saccharomyces cerevisiae* (Asghari-Paskiabi et al., 2019). The microorganisms converted selenium ions to elemental selenium in order to prevent the ions toxicity. It is supposed that in the defense against selenium ions toxicity, sulfite reductase (SiR) reduced soluble selenium ions of selenous acid to insoluble elemental selenium. After supersaturation of the atoms under ambient conditions, the nucleus would emerge. The nucleus released maximum amount of energy to become stable and crystallized (Thanh et al., 2014).

**FIGURE 1:**
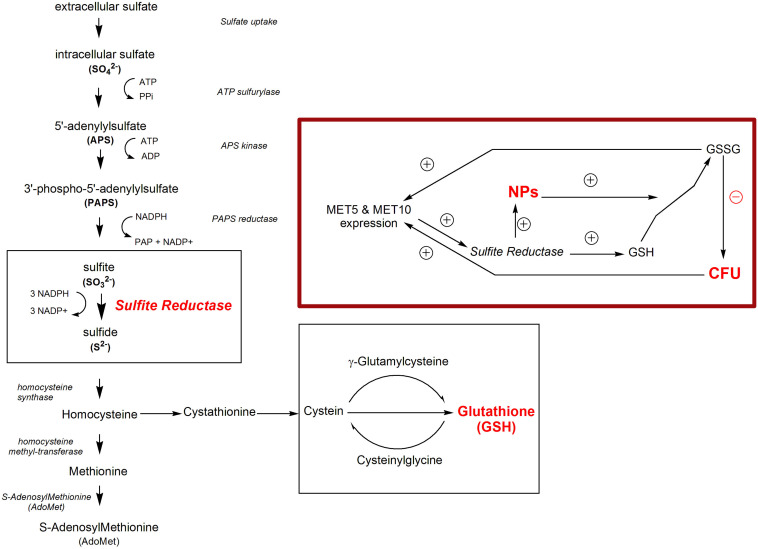
Sulfur pathway in *S. cerevisiae*. The inset shows the correlation between NPs synthesis, sulfite reductase activity and the expression of sulfite reductase main genes, and *MET5* and *MET10*.

In sulfur cycle, the microorganism produces antioxidants, which are needed to tolerate toxic ions and NPs (Grant and Dawes, 1996). SiR is the main enzyme in sulfur cycle for the production of sulfide from sulfite (Elskens et al., 1991; Thomas and Surdin-Kerjan, 1997). On the one hand, this enzyme is responsible for the reduction of ions and NPs synthesis, and on the other hand, it is responsible for the production of antioxidants against toxic materials, such as the NPs. Thus, it was motivating to know the enzyme activity and expression of its genes as well as the rate of population growth before, during, and after the production of NPs. In all organisms, sulfur exists in amino acids cysteine and methionine as well as in coenzymes, metabolites, and chemical structure; however, fungi obtain sulfur from inorganic sulfate species present in nature. In sulfate assimilation process, sulfate is incorporated into cysteine (Leustek et al., 2000; Kopriva, 2006). In this process, sulfate is activated in two steps into adenosine 5’phosphosulfate (APS) or 3’phosphoadenosine 5’phosphosulfate (PAPS), each of which is reduced to sulfite by APS or PAPS reductase (PAPR). Sulfite is reduced to sulfide by SiR, which is composed of α (116 kDa) and β (167 kDa) subunits (Figure 1; Patron et al., 2008). Moreover, in *S. cerevisiae*, the 6-electron-sulfite is reduced to sulfide ion by sulfite reductase (EC 1.8.1.2) catalysis (Jiranek et al., 1996). Sulfite reductase is known to have the main role in the biosynthesis of sulfide-metal NPs (Senapati et al., 2014).

In this research, sodium sulfite/selenous acid as precursors, were added to *S. cerevisiae* culture medium in order to obtain selenium sulfide NPs. Thus, there was a shortcut in the cells’ sulfur cycle, where the sulfate was omitted, and the cycle started from sulfite. In this study, we aimed to satisfy a curiosity on how the interaction of a microorganism with selenium ions and their conversion to NPs affect the generating fungus itself, e.g., the changes in gene expression, enzymatic system, and finally its population as a sign of toxicity. To this end, we quantified the population profile of the *S. cerevisiae* cells in terms of colony forming units (CFU) in the presence and absence of the selenium substrate. Then, the variation of sulfite reductase activity, the expression of two main genes of sulfite reductase (*MET5* and *MET10*; dependent variables), and concentration of sulfite and selenium (independent variables) in the culture medium, were evaluated. Finally, the effects of the independent variables on the *S. cerevisiae* cell population as well as the population growth on some dependent variables were modeled using a path analysis approach.

## Materials and Methods

### Materials

*Saccharomyces cerevisiae* PTCC 5052 was obtained from Persian Type Culture Collection (PTCC), Iranian Research Organization for Science and Technology, Tehran, Iran. Orthophosphoric acid, glucose, potassium dihydrogen phosphate, and di-potassium hydrogen phosphate and anhydrous Iron (III) chloride were purchased from Merck Chemicals Co. (Darmstadt, Germany). Sabouraud dextrose agar (SDA), peptone and yeast extract were obtained from MicroMedia, Hungary. Hydrogen sulfide, sodium sulfite, N,N-dimethyl-*p*-phenylenediamine dihydrochloride, glucose-6-phosphate dehydrogenase (G-6-P-DH), Ethylene glycol-bis(β-aminoethyl ether)-N,N,N’,N’-tetraacetic acid (EGTA) tetrasodium salt, glucose-6-phosphate (G-6-P), Coomassie Brilliant Blue G-250, bovine serum albumin (BSA), Roswell Park Memorial Institute (RPMI) 1640, selenous acid, and sodium sulfite were all obtained from Sigma-Aldrich (Milwaukee, United States) and used as received. RNA extraction kit was purchased from Bio Basic Inc., Canada. cDNA synthesis kit, reverted reverse transcriptase was obtained from Thermo Scientific, Lithuania. Sulfite measurement kit was obtained from VAHEB chemical company, Iran. Real-Time Master Mix was purchased from BioFACT^TM^, South Korea. All of the chemicals were of analytical or reagent grade and used as received without further purification. Distilled water was prepared in-house by reverse osmosis technique.

### Cell Culture and Count

Minimum inhibitory concentration (MIC) of the precursor which *S. cerevisiae* could not tolerate, was determined beforehand according to Wayne, 2008. First, a 0.5 McFarland suspension (1.2 × 10^6^ cells/mL) was made in distilled water which was diluted ten times in RPMI 1640. Aliquots of 100 μL of the cell suspension were dispensed in wells of a microtiter plate and 100 μL of the salt i.e., sodium sulfite/selenous acid (50/50 w/w) in concentrations of 3, 1.5, 0.75, 0.375, 0.187, 0.093, 0.046, and 0.023 mM were added. All experiments were carried out in three sets of replicates for 24 h at 35°C.

*Saccharomyces cerevisiae* yeast cells were harvested from 24-h solid cultures of SDA and inoculated into liquid medium of GYP (glucose 2%, yeast extract 1%, and peptone 2%) at a concentration of 10^6^ cells/mL. Five sets of experiments, were carried out (*n* = 3) in parallel at five different incubation times. The first set of experiments (negative control groups) was free of precursors. First, the salts, i.e., sodium sulfite/selenous acid (50/50 w/w, 1 mM), were added to the 24-h yeast cells in the liquid culture medium and then the medium was incubated for 100, 200, 300, and 400 min under orbital shaking at 180 rpm using a shaking incubator (LabTech DAIHAN LABTECH CO., LTD., LSI-3016 R, South Korea) at 35°C. Since we optimized the concentrations of selenous acid/selenium sulfite (50:50 w/w) at levels that do not affect the growth of *S. cerevisiae* at 35°C according to CLSI documents, we used this temperature for the yeast growth in all the experiments throughout the work even if the temperature is not the optimal for the yeast cells.

**FIGURE 2:**
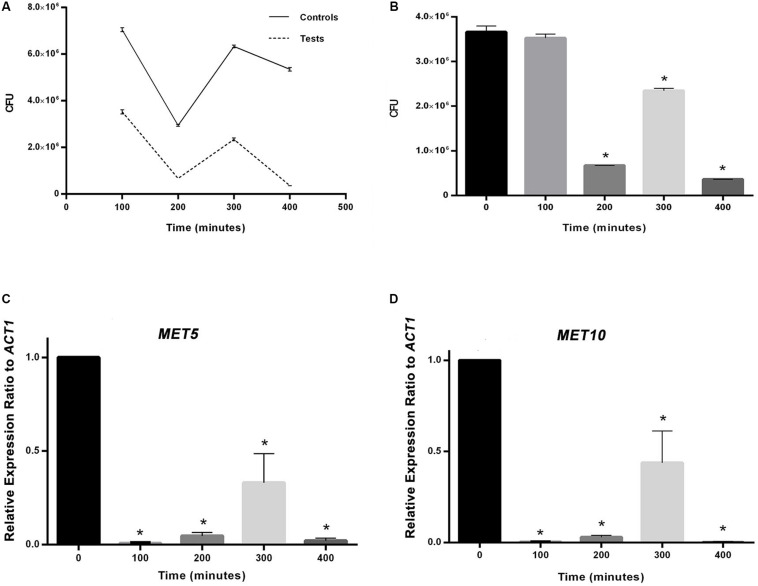
**(A)** The effect of experimental conditions on *S. cerevisiae* population in comparison with the control. **(B)** Comparison between population of *S. cerevisiae* in time point zero and following time points. **(C)** and **(D)** Comparison between relative expression ratio of *MET5* and *MET10* genes to *ACT1* in time point zero and following time points. Asterisks represent significant differences at *p* < 0.05.

The intervals were selected based on the time required for cells to proliferate (Kim and Wang, 1989), and extended to 400 min. At each time interval, corresponding tests were performed on the samples, as schematically shown in Figure 2 The first time point (*t* = 0 min) represents another negative control group, where no precursor salt was added. For CFU count, *S. cerevisiae* suspensions were first diluted 1/100 and then 1/10000 in deionized water. 50 μL of each dilution was uniformly spread over a 6-mm solid SDA supplemented with chloramphenicol (0.05 g/L) and incubated at 35°C. CFU were counted after 24 h and calculated based on dilution factors.

### *MET5* and *MET10* Gene Expression

*MET5* and *MET10* gene expression analysis was carried out at 5 time points. *ACT1* gene was considered as a reference gene. All works were performed on ice (*T* = 4°C). Cells were separated from liquid culture medium by centrifugation at 15000 × *g* (*T* = 4°C) for 20 min. Then, the cells were washed immediately using normal saline solution. Cells were lysed using Rlysis-FG buffer as a medium containing 7 to 8 glass beads (diameter = 0.5 mm) in a 1.5 mL RNase-free microtube containing *S. cerevisiae* cells. The vortex mixing was performed on ice 5 times (each 60 s) with 60 s intervals. RNA isolation was performed using the mentioned kit according to the manufacturer’s instruction. The obtained extract was subjected to optical density measurement using a NanoDrop spectrophotometer (ND-1000, United States) and the amounts of RNA were normalized before the next step.

cDNA was synthesized by a Thermo Scientific Reverse Transcriptase kit using random hexamer primers. The procedure was performed exactly according to the manufacturer’s instruction in a volume of 1000 μg of RNA.

For PCR amplification, 1 μL of cDNA was added to 1 μL of specific primers (Table 1) in 10 μL of 2X Real-Time PCR Master Mix (BioFACT^TM^, South Korea) and the whole solution reached to 20 μL (final reaction volume) using deionized water. A Corbett Research, RG-6000 Real Time PCR thermocycler (Australia) was used for all amplifications. The initial step took 2 min at 94°C, followed by 30 cycles of 94°C for 30 s, 60°C for 30 s, and 72°C for 30 s. All samples were analyzed in triplicate. A standard curve was plotted for *MET10* primers by serial dilution of the cDNA solution. The coefficient of efficiency (E) was calculated based on the following equation:

**TABLE 1:** Sequences of the qPCR primers.

Genes	Nucleotides	Accession number
*MET5*	F-5’-GAGCCTGAGAGACCATTT-3’	CP020234.1
	R-5’-GAGGCAAATCTGGTGTAT-3’	
*MET10*	F-5’-AGAGGATTTGGTTACTCC-3’	CP020213.1
	R-5’-AGTTCCTCAAGAGATGGG-3’	
*ACT1*	F-5’-GGATCTTCTACTACATCAGC-3’	CP020179.1
	R-5’-CACATACCAGAACCGTTATC-3’	

(1) $$\text{E}=\left[10^{-1/\text{slope}}\right]\,-1$$

To evaluate the variability of the genes’ expression, the obtained delta cycle threshold (CT) were analyzed by ΔΔCT method via:

(2) $$\Delta \,\text{CT}={\text{CT}}_{\text{target}}-{\mathrm{CT}}_{\text{reference}}$$

(3) $$\Delta \,\Delta \,\text{CT}=\Delta \,{\text{CT}}_{\text{sample}}-\Delta \,{\text{CT}}_{\text{control}}$$

Here, the control is the sample of zero time.

### Sulfite Ion Concentration

The content of cultures at all 5 time intervals was centrifuged at 15000 × *g* for 10 min and the supernatant was passed through 0.22 μm syringe head filters. The sulfite content of the filtrate was measured by a well-known iodometric titration method using a kit (Sroysee et al., 2016). To this end, 10 mL of the filtrate was added to a plastic test tube, where 5 drops of sulfuric acid was added, and followed by 3 drops of starch. The combination was mixed by shaking the tube in each step. At the end, titration was performed by adding potassium iodate and potassium iodide until the appearance of a blue iodine color.

### Selenium Concentration

Measurement of Selenium was performed on the filtrates, as previously explained under sulfite ion measurement. The measurements were performed using an atomic absorption instrument (Thermo Jarrell Ash, Smith-Hieftje 22), which was operated at a wavelength of 196 nm, bandpass of 2 nm, lamp current of 5 mA, and sensitivity of 0.4 ppm. Standard solutions of selenous acid were prepared in the range of 0 to 200 mg L^–1^. The selenium was measured in the samples and the concentrations were calculated based on the standard curve of selenium.

### Total Protein Concentration

Total protein content was measured using Bradford method (Bradford, 1976). First, the standard curve was plotted by BSA. Then, protein measurements were performed on extracts isolated from *S. cerevisiae* cells before dialysis.

The Bradford reaction was initiated by first adding 100 mg of Coomassie Brilliant Blue G-250 dye to 50 mL of ethanol 95% solution and then to 100 mL of orthophosphoric acid 85% (w/v) solution. Then, the volume of the solution was brought to one liter using distilled water. Optical absorbance of standard samples and the test specimens, were recorded with a Perkin Elmer UV/Vis spectrophotometer at a wavelength of 595 nm, 2 (to max 20) min after the Bradford reaction. Protein concentrations were calculated using a standard curve.

### Sulfite Reductase Activity

Sulfite reductase activity was measured according to the previously reported methods with slight modifications (Jiranek et al., 1995, 1996). For this purpose, first the following solutions were made:

Buffer A solution: Phosphate buffer (0.25 M, pH 7.3) supplemented with EGTA (1 mM).
Buffer B solution: Buffer A solution supplemented with glycerol (20% V/V).
Reagent C solution: A solution comprising G-6-P (1.7 mM), MgCl_2_ (1 mM), Na_2_SO_3_ (0.1 mM), NADP (0.1 mM), and G-6-P-DH (166 U/L), which was freshly prepared.
Reagent D solution: First a stock solution of N,N-dimethyl-*p*-phenylenediamine dihydrochloride (0.75 M) was made in sulfuric acid (11.3 M). Stock solution (250 μL) was added to a solution comprising sulfuric acid (9 M, 4.75 mL) and ferrous chloride (FeCl_3_, 555 μL, and 40% W/V in sulfuric acid 9 M). Reagent D solution was freshly prepared.

Cells were harvested by centrifugation (11300 rpm, 15 min at 4°C) and washed twice with cold buffer A solution. Then, 1 mL of cold buffer B solution was added per every 200 mg of the wet cells. To break the cell walls and lyse them, a freeze-thaw method was applied on the samples for 5 cycles, comprising immersion in liquid nitrogen (10 s), and consequent thawing in ambient conditions for the same time duration. Then, glass beads (Weight ≅ 10 *g*, diameter = 0.5 and 1 mm in mixture), were added to the suspension and the whole was subjected to vortexing (*t* = 30 s) – resting on ice (*t* = 60 s) in several cycles. The lysis operations were conducted until assurance of lysis of more than 90% of the cells based on optical microscopy results. The crude extract was centrifuged at 40000 × *g* at 4°C for 75 min. The transparent supernatant was dialyzed twice through a 12 KD dialysis membrane against fresh buffer B and incubated at 4°C for 75 min.

Reagent C (1.5 mL) and cell extract (200 μL), were added to disposable test tubes with rubber caps and the total volume was adjusted to 2 mL using buffer B solution. The tubes were gently inverted several times and incubated for 1 h at 30°C. Then, reagent D (150 μL), was injected into the tubes through the rubber cap using a syringe. The tubes were shaken vigorously and incubated for another hour at 30°C. Then, the tubes were centrifuged at 12000 × *g* for 5 min. Optical density of the supernatant was measured at 619 nm. Blank and standard samples were prepared as well except that instead of the cells extract, deionized water and H_2_S standard were determined, respectively. H_2_S concentration was calculated by standard curve. The enzyme activity was normalized based on the total protein concentration.

### Statistical Analysis

To assess the difference in yeast population growth in the presence and absence of the abiotic variables, Mann-Whitney *U* test was used (Nachar, 2008). The effect of independent variables on the yeast population growth, as well as the activity of sulfite reductase, and expression of *MET5* and *MET10* genes was assessed using path analysis method as explained by Wright (1921).

In this technique, correlation coefficient between all subsets of variables was estimated. Correlation coefficients indicate the degree to which population growth is influenced by each independent variable. The results were grouped into a causal model, showing possible pathways of independent-dependant correlation. As the dependant variables were not completely explained by independent variables, the unexplained variation was showed as the residual error in the model. Correlations were considered as statistically significant at 0.05 levels. Analysis was performed in Stata software (version 14).

## Results

First, the growth of *S. cerevisiae* yeast cells under experimental conditions was compared with their counterparts in the control group. To this end, CFU counting was performed at 5 time intervals for *S. cerevisiae* yeast cells with and without salt treatment. Then, sulfite reductase activity of the cells, expression of *MET5* and *MET10* genes, and sulfite and selenium concentrations of the culture medium, were measured (Figure 2). The intervals were selected based on the time required for cells to proliferate (Kim and Wang, 1989), and extended to 400 min. NPs formed after 100 min and we were sure of their production after 240 min.

### Colony Forming Unit Measurements and Quantitative Measurement of Sulfite Reductase *MET5* and *MET10* Expressions of *S. cerevisiae* in Experimental Conditions

In MIC analysis no growth inhibition was seen from 0.0234 to 3 mM concentration of the precursor (Supplementary Figure S1). As it can be seen in Figure 2B, CFU values obtained at all time points, were less than their counterparts in the control group (*p* < 0.05), as far as the CFU count was tending toward zero at the last time interval in contrast to the control group. Moreover, the CFU at all time points, except the first one, was significantly different from the time point zero.

The variations occurring in *MET5* and *MET10* genes expression are displayed in Figures 2C,D. The rate of genes expression significantly decreased from the first measurement time point after adding the substrates. The mean expression of both genes had a rise at 300 min and a sharp drop at 400 min. The expression of the genes in all the following four points, was significantly different from the point zero.

### NADPH-Dependent Sulfite Reductase Activity in *S. cerevisiae* Cell Extracts and Sulfite and Selenium Measurements

**FIGURE 3:**
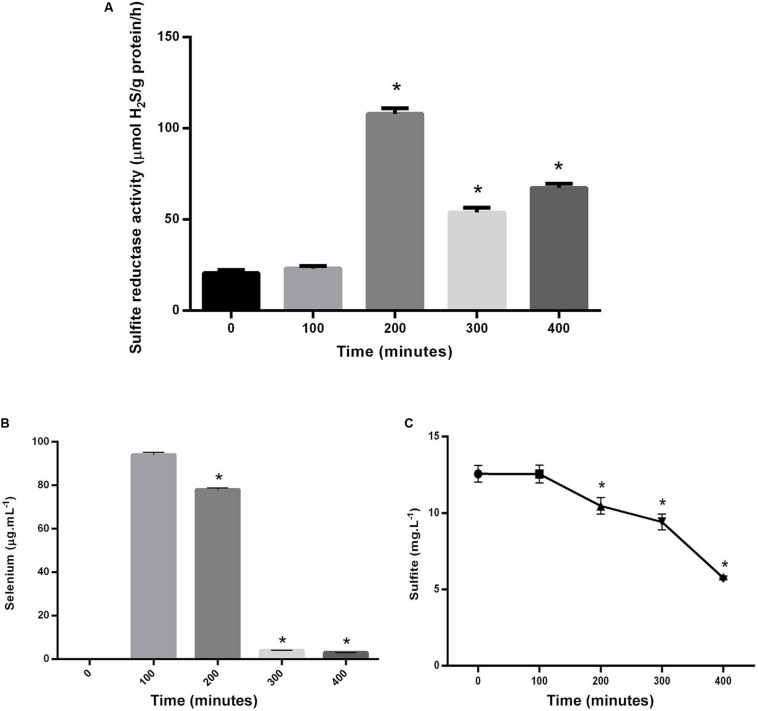
**(A)** Comparison between the variation of sulfite reductase activity in *S. cerevisiae* in time point zero and experiments in following time points. **(B)** Comparison between selenium levels in the supernatant of control and experiments in following time points. **(C)** Comparison between sulfite levels in the supernatant of control and experiments in following time points. Asterisks represent significant differences at *p* < 0.05.

According to the results (Figure 3), the changes in sulfite reductase activity at the time points 2 to 4, were significantly different from the time point zero. Fluctuation of the sulfite reductase activity was opposite to their counterparts’ time points in the CFU measurements. For example, at time point 2, in which the enzyme activity was at its highest level, the CFU count was at its lowest.

The fluctuations of cells’ consumption of sulfite and selenium were shown in Figures 3A,B. The rate of both selenium and sulfite tended to decrease over time. Their amounts at the time points 2, 3, and 4, were significantly different from the time point 1, when the substrates were added to the culture media of *S. cerevisiae*. The amount of selenium in the filtrate was indicative of its consumption level by cells or the amount of NPs produced inside the cells. Therefore, there was a direct relationship between selenium decrease in filtrate solution and NPs increase in *S. cerevisiae* cells. The amount of sulfite in the filtrate was also measured. Although, it decreased over time, it did not reach zero or near zero at the last measured time point, as it was not zero, even when the salts had not been added at the first time point.

### Correlations Between the Measured Variables

**FIGURE 4:**
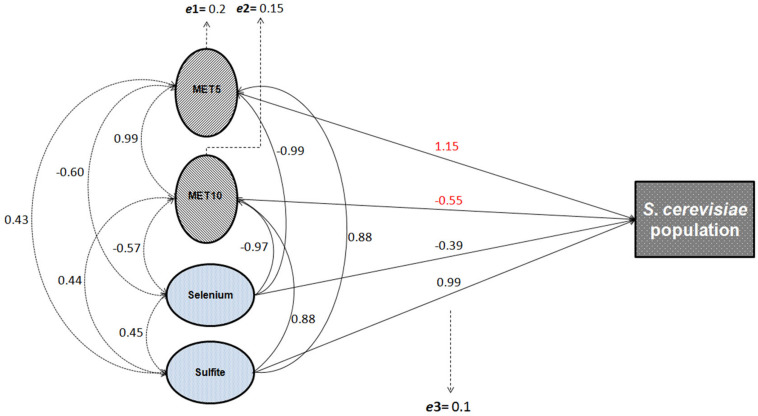
General diagram of the possible relationships between abiotic and gene expression variables and the population growth of *S. cerevisiae*. Solid arrows show the direct effects of independent variables on the dependent variables (*S. cerevisiae* population growth, gene expression, and sulfite reductase activity). Dashed arrows show the indirect effects, i.e., the correlation coefficients among variables. The correlation and path coefficients for these relationships are also shown in the Supplementary Tables S1, S2; *e*1: residual variable of the model assessing the direct effect of sulfite and selenium on *MET5* expression; e2: residual variable of the model assessing the direct effect of sulfite and selenium on *MET10* expression; and e3: residual variable of the model assessing the direct effect of sulfite, selenium, and the expression of *MET5* and *MET10* on yeast population growth.

The correlation matrix showing all possible correlations between all the independent and dependent variables is presented in Supplementary Table S1. Our results showed that sulfite and selenium concentrations in filtrate solutions were significantly correlated with population growth of *S. cerevisiae* (*p* = 0.021 and *p* = 0.109, respectively; Supplementary Table S2), and the expression of *MET5* (*p* = 0.019, and *p* = 0.015, respectively) and *MET10* (*p* = 0.044, and *p* = 0.037, respectively; Supplementary Table S3), but had no significant correlation with sulfite reductase activity. Sulfite increased the population growth and *MET5* and *MET10* expression, while according to statistical findings, the ratio of selenium in the solution at each time point (which due to the consumption of yeast cells was gradually decreasing compared to the initial amount) was inversely related to population growth and *MET5* and *MET10* expression at the same point. Both abiotic variables (sulfite and selenium) explained 0.97, 0.98, and 0.94% of the total variation of population growth and expression of *MET5* and *MET10* genes, respectively (Supplementary Tables S2, S3). This finding is highly useful in predicting gene expression and understanding the mechanism of the expression. Direct and indirect effects of selenium on the population growth, were not the same. Therefore, the effect of this variable on yeast population growth, was considered to be indirect, probably through changes in the rate of NPs synthesis. Interestingly, when the samples were grouped and analyzed by considering all the variables, including abiotic variables, sulfite reductase activity, and genes’ expression, all assessed variables explained more than 99% of the variations observed in the growth of *S. cerevisiae*. Although, the variables were not statistically significant, probably due to small sample size. CFU count revealed a significant negative effect on the sulfite reductase activity, but no significant effect on the expression of *MET5* and *MET10* genes, probably due to the lack of power (Supplementary Table S4). Sulfite reductase activity also had no significant effect on the expression of *MET5* and *MET10* genes (Supplementary Table S5). A general correlation diagram showing all possible relationships between independent variables (abiotic variables) and dependent variables (gene expression, sulfite reductase activity, and *S. cerevisiae* population), is given in Figure 4.

## Discussion

In our previous study, the cell wall and cell membrane of the yeast cells were quite dark and the NPs were observable inside the cells, indicating that *S. cerevisiae* converted selenium salts into selenium sulfide NPs inside the cells (Asghari-Paskiabi et al., 2019). Kasemets et al. (2009) have evaluated the toxicity of CuO NPs to *S. cerevisiae*. In comparison with bulk CuO, the NPs were 62–94 times more toxic after 8 and 24 h of exposure. It was not supposed that NPs were internalized. They attributed half of toxicity to the solubilized Cu ions. So solubilized Cu ions was not the sole reason of toxicity (Kasemets et al., 2009). Oxidative stress, mediated by reactive oxygen species (ROS) is considered as the main mechanism of NPs toxicities (Shvedova et al., 2005). Cellular carbohydrates, lipids, DNA, and proteins can be damaged by ROS followed by oxidative stress and inflammation (Kasemets et al., 2009).

In a study by Li et al. (2017), it was revealed that *Sclerotinia homoeocarpa* cells can internalize ZnO and Ag NPs or their ions, but the mentioned NPs or ions can cause oxidative damage in the cells. The ions may also accumulate inside the cell, which is a reducing environment that can reduce the ions to NPs, damaging the cells in another way. They found that after exposure to the NPs, two stress response genes, GST (*Shgst*1), and superoxide dismutase 2 (*Sh*SOD2), were significantly upregulated. They believed that the induced expression of *Shgt1* gene was probably due to glutathione (GSH) depletion (Li et al., 2017; Figure 1). GSH reduces metal ions toxicity in the cells by binding to them. Also, Huerta-García et al. (2014) indicated that mRNA expression of *SOD2* was induced by NPs in glial cells. They believed that some changes in the redox state and initial rise in the ROS, can be the induction factors for expression of the genes responsible for the response against oxidative stress (Huerta-García et al., 2014). Hanagata et al. (2011) studied genome expression of human lung epithelial A549 cells exposed to CuO NPs. It was observed that CuO NPs and its ions, as ROS, and caused SOD2 upregulation (Hanagata et al., 2011). Also, cadmium increased ROS production and induced catalase activity. A Cd-dependent depletion of intracellular GSH and a rise in its external concentration, were seen concomitantly (Pacheco et al., 2008).

**FIGURE 5:**
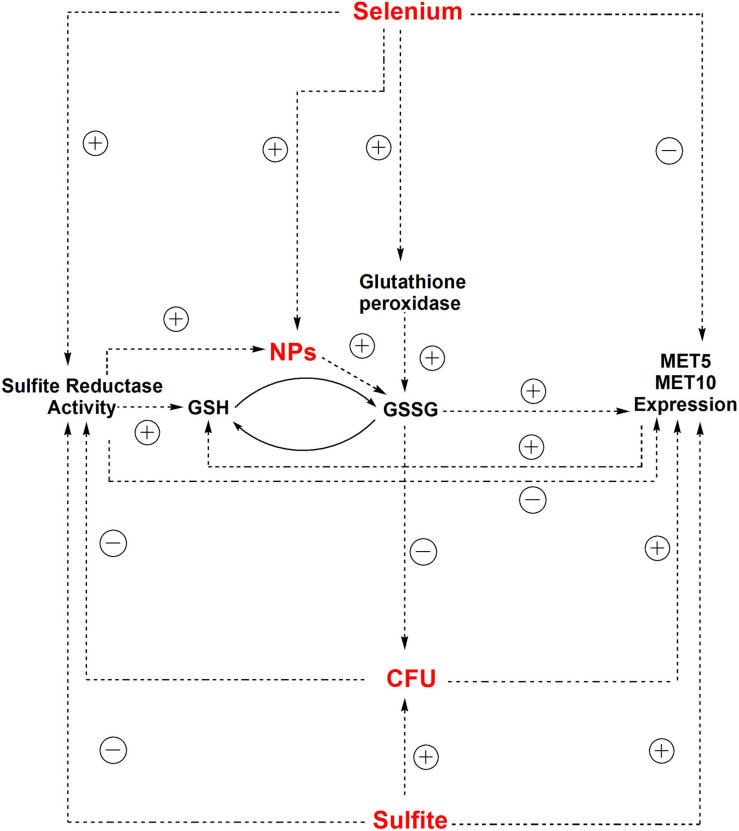
The correlation between independent parameters (selenium and sulfite) and dependent parameters (CFU counts, *MET5*/*MET10* expression, and sulfite reductase activity) and other relationships between dependent and independent parameters in experimental condition of NPs synthesis.

Depletion of GSH, one of the earliest antioxidant molecules in most of the cells, is the main mechanism of metal toxicity (Stohs and Bagchi, 1995). The same happened in human cells after exposure to high doses of Zn ferrite NPs (Alhadlaq et al., 2015). Piao et al. (2011) reported that Ag NPs inhibited GSH-synthesizing enzymes (GCLC and GSS). GSH levels decreased, leading to ROS generation in human Chang liver cells (Piao et al., 2011). According to a study by Ma et al. (2015), exposure to Ag NPs caused reduction of GSH levels in *Crambe abyssinica* plant, which was attributed to H_2_O_2_ and Ag ion detoxification through oxidation of GSH to GSSG (Ma et al., 2015). It can be supposed that reduction of selenium ions to selenium sulfide NPs altered the redox state of *S. cerevisiae* cells and led to an increased activity of ROS. Also, the presence of the NPs depleted the cells from GSH, which is one of the initial defense factors against toxicity of the oxidizing agents and metals (Figure 5).

The yeast cells can absorb selenium through their hydrophobic cell wall structure (Kordialik-Bogacka, 2011). Selenium binds to polysaccharide in the cell wall and its binding rate directly depends on the selenium concentration in the culture media (Kieliszek et al., 2015). Selenium exists in GSH peroxidase structure (Galiazzo et al., 1987), so that selenium treatment significantly increase GSH peroxidase activity, and supporting the role of selenium in enzymatic defense mechanisms of yeasts against oxidative stress (Grant et al., 1996; Kaur and Bansal, 2006). GSH peroxidase activity requests GSH as a substrate, but GSH synthesis itself depends on sulfite reductase activity (Hansen and Johannesen, 2000), thus, in this study sulfite reductase activity increased to supply the additional need of GSH in response to the risk of NPs (Figure 5). This is in agreement with findings of Kaur and Bansal (2006) study, indicating that increased GSH peroxidase activity results in the need for more amount of GSH, which is produced by sulfite reductase (Kaur and Bansal, 2006).

The correlation between gene expression and the cell growth is in agreement with what Scott et al. (2010) reported in *Escherichia coli*. They deduced that chloramphenicol-induced reduction of translational capacity, was compensated by an increase in the RNA/protein ratio through inhibiting the repression of rRNA synthesis (Scott et al., 2010). Inverse correlation between selenium concentration and expression of *MET5* and *MET10* genes, was due to the reduction of microorganism population in the presence of selenium based-NPs (Figure 5), since there was a positive direct relationship between expression of *MET5* and *MET10* genes and cells growth.

According to the statistical analysis, sulfite reductase activity had a significant (*p* = 0.047) negative effect on the growth of the cells. It is assumed that the decrease in *S. cerevisiae* cells growth was followed by a corresponding increase in sulfite reductase activity, which in turn caused more consumption of sulfite from the environment. The statistical analysis showed that there was a direct significant correlation between growth of the cells and the amount of sulfite in *S. cerevisiae* cells culture medium (*p* = 0.021). Sulfite had a positive significant effect on *MET5*/*MET10* genes expression (*p* = 0.019, *p* = 0.044), but there was a non-significant negative correlation between the sulfite and sulfite reductase activity. It meant that sulfite played a role in control of the expression of *MET5* and *MET10* and probably not sulfite reductase activity (Figure 5).

*SSU1* encodes a plasma membrane protein that plays a role in sulfite metabolism. *SSU1* mutation causes sulfite sensitivity and its over-expression results in resistance to sulfite, supporting its role in detoxification. Also, resistance to sulfite needs a transcriptional activator from dominant allele of SSU1, called five zinc finger (FZF1). Park and Bakalinsky (2000) showed that ssulp protein was involved in sulfite efflux and FZF1-4 caused resistance to sulfite by an increase in the activators of SSU1. They observed that sulfite reductase did not consume sulfite, therefore, a major part of the initial sulfite content in the culture medium (more than 95%), was recovered in the form of intra and extra-cellular sulfite at the end of experiment, and up to 4 mM sodium sulfite did not alter viability of the cells according to the sulfite accumulation test (Park and Bakalinsky, 2000). Most *S. cerevisiae* species produce sulfite. Dott and Trüper (1976) categorized them in groups of “low” and “high,” depending on the amount of their sulfite production (Dott and Trüper, 1976). This can explain the reason why the level of sulfite in the *S. cerevisiae* culture medium, was not significant, either at the time zero before adding sulfite or 400 min after adding it.

Schimz (1980) investigated the effect of sodium sulfite (1 mM) on *S. cerevisiae* up to 160 h and observed approximately no effect on its growth. They assumed that *S. cerevisiae* cells resistance to sulfite depended on pH, temperature, physiological conditions of the cells, and incubation time. For example, acidic pH caused a decrease in resistance and 0.05 mM sulfite in comparison with 10 mM sulfite resulted in more resistant cells. Also, cells were more resistant at 18°C compared to 28°C (Schimz, 1980). In fact different microorganisms have various pH range for oxidation of sulfur (Plumb et al., 2008), which is an acid-producing reaction (Liang et al., 2020). These findings are in agreement with our results that there was a significant positive relationship between growth of cells and sulfite level in the medium.

In this series of experiments, growth of the cells indicated a significant decrease compared to the time point zero, except time point 1 that was the beginning of NPs production. Population growth monitoring was done during NPs synthesis inside *S. cerevisiae* cells. By more production of NPs, more reduction in *S. cerevisiae* population happened. While, sulfite had a positive significant effect on the cells growth, the relationship between selenium and population was indirect. It can be concluded that the produced selenium sulfide NPs, caused this decrease in the growth of cells. However, the ability of selenium NPs in modulation of antioxidative defense system has made them less genotoxic in mice (Bhattacharjee et al., 2019) and less toxic in rats (Menon et al., 2019) compared to other chemical forms of selenium. Figure 5 illustrates the inter-intra relationship between all parameters and cell population.

## Conclusion

Biosynthesis of selenium sulfide NPs by *S. cerevisiae* resulted in a significant decrease in CFU counts within 400 min. The path analysis model revealed that the expression of the main genes of sulfite reductase (*MET5* and *MET10*), and thus, the enzyme responsible for converting the substrates to selenium sulfide NPs, conformed to the CFU counts of *S. cerevisiae* in 400 min. The activity of sulfite reductase showed an inverse correlation with CFU counts of the *S. cerevisiae* cells. It means that more activity of the enzyme was observed with lower levels of cell growth. There was a positive correlation between the levels of sulfite in *S. cerevisiae* medium and the CFU counts, while a negative correlation was observed between the amount of selenium and CFU count. The obtained NPs were the factors that damaged *S. cerevisiae* cells. Hopefully, this phenomenon can be used to eliminate the toxic dose of selenium ions or harmful microorganism from the natural sources of life in addition to green synthesis of selenium sulfide NPs.

## Data Availability Statement

All datasets generated for this study are included in the article/Supplementary Material.

## Author Contributions

FA-P, MI, and MR-A designed the experiments. SE did the modeling. MI, HR-T, and MR-A supervised the study. FA-P performed the experiments and wrote the manuscript. All authors approved the final version of manuscript.

## Conflict of Interest

The authors declare that the research was conducted in the absence of any commercial or financial relationships that could be construed as a potential conflict of interest.
