# Supplementary material for: Population Kinetics and Mechanistic Aspects of Saccharomyces cerevisiae Growth in Relation to Selenium Sulfide Nanoparticle Synthesis
Source: Front Microbiol. 2020 May 21;11:1019. doi: 10.3389/fmicb.2020.01019 (PMC7253647; doi:10.3389/fmicb.2020.01019)
Supplement: Supplementary file 1 [file Data_Sheet_1.DOC]

**Population Kinetics and Mechanistic Aspects of *Saccharomyces cerevisiae* Growth in Relation to Selenium Sulfide Nanoparticle Synthesis**

Farnoush Asghari-Paskiabi^1, 2^, Mohammad Imani^3,**^, Sana Eybpoosh^4^, Hashem Rafii-Tabar^1^, Mehdi Razzaghi-Abyaneh^2,*^

*^1^Department of Medical Physics and Biomedical Engineering, School of Medicine, Shahid Beheshti University of Medical Science, Tehran, Iran*

*^2^Department of Mycology, Pasteur Institute of Iran, Tehran 13164, Iran*

*^3^Novel Drug Delivery Systems Department, Iran Polymer and Petrochemical Institute, P.O. Box 14965/115, Tehran, Iran.*

*^4^Department of Epidemiology and Biostatistics, Research Centre for Emerging and Reemerging infectious diseases, Pasteur Institute of Iran, Tehran, Iran.*

**Correspondence and reprints:**

Mehdi Razzaghi-Abyaneh* Mohammad Imani**

Tel: +98-21-64112804 Tel: +98-21-4866 2456

Fax: +98-21-66465132 Fax: +98-21-4458 0021

E-mail: [mrab442@yahoo.com](mailto:mrab442@yahoo.com) & Email: [M.Imani@ippi.ac.ir](mailto:M.Imani@ippi.ac.ir)

[mrab442@pasteur.ac.ir](mailto:mrab442@pasteur.ac.ir)

https://orcid.org/0000-0001-8217-399X


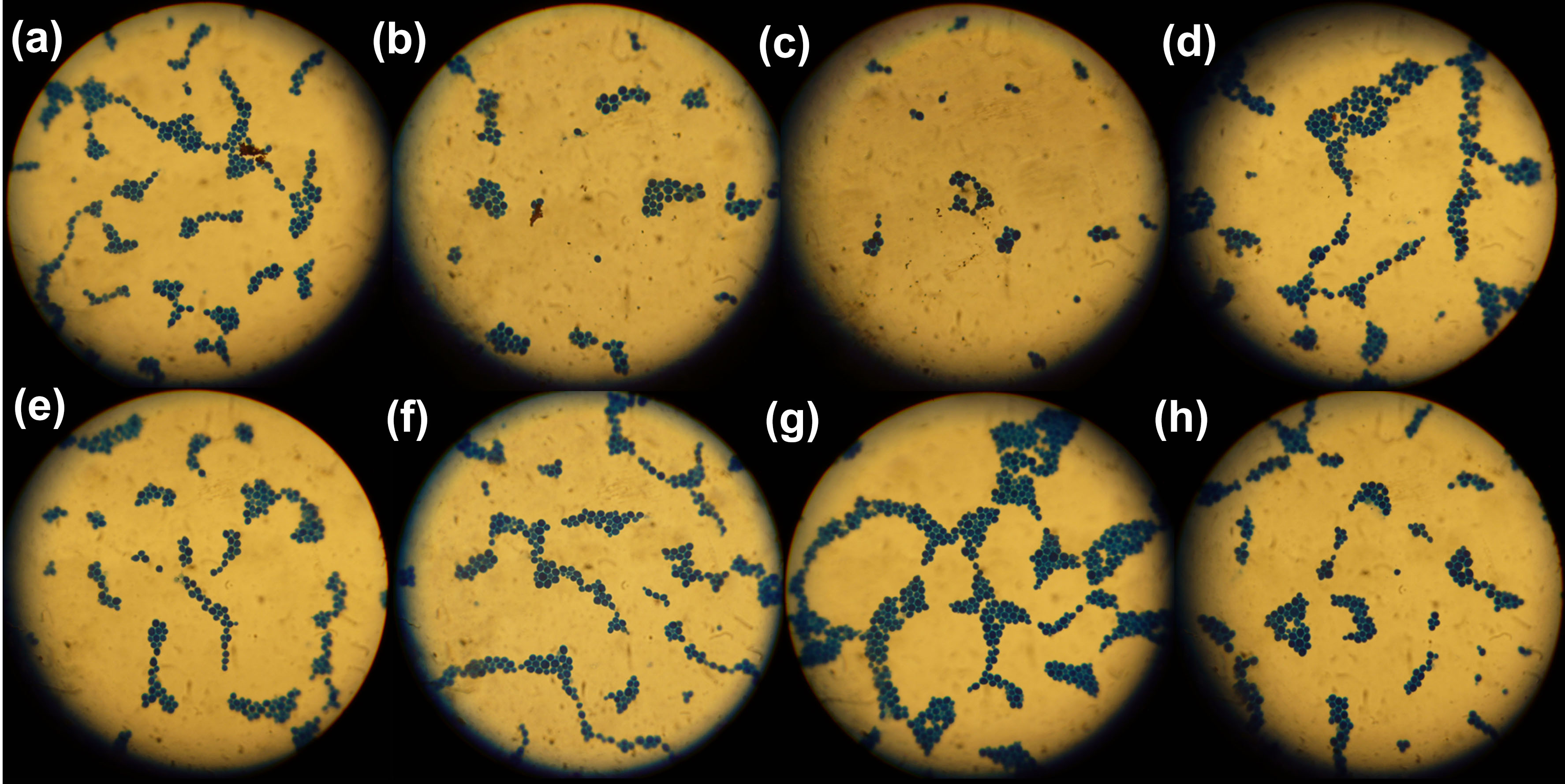


**Fig. S1.** Optical microscopy images of cells in MIC test (1000 ×). The specimens were stained by methylene blue. The concentrations of sodium sulfite/selenous acid (50/50 w/w) were: (a): 3 mM; (b): 1.5 mM; (c): 0.75 mM; (d): 0.375 mM; (e): 0.187 mM; (f): 0.093 mM; (g): 0.046 mM and (h): 0.023 mM.

S_2_: Selenous acid/sodium sulfite treated *S. cerevisiae* cells

**Table S1**. Indirect effect (correlation matrix) between variables measured in this study

|  | **CFU** | **Enzyme** | **MET5** | **MET10** | **Sulfite** |
| --- | --- | --- | --- | --- | --- |
| **Sulfite reductase activity** | -0.88 |  |  |  |  |
| **MET5 expression** | 0.60 | -0.53 |  |  |  |
| **MET10 expression** | 0.59 | -0.52 | 0.99 |  |  |
| **Sulfite** | 0.82 | -0.49 | 0.43 | 0.44 |  |
| **Selenium** | 0.062 | 0.19 | -0.60 | -0.57 | 0.45 |

**Table S2. Direct effect (correlation coefficients) of abiotic variables measured on *S. cerevisiae* population growth**

|  | **Standardized correlation coefficient (*β*)** | ***P* value** |
| --- | --- | --- |
| **CFU** |  |  |
| Sulfite (μg.mL^-1^) | 4.12 | 0.021 |
| Selenium (μg.mL^-1^) | -4.0 | 0.109 |
| Coefficient of determination (*R^2^*) | 0.97 | - |
| Residual variable (ᵋ) | 0.10 | - |

**Table S3. Direct effect (correlation coefficients) of abiotic variables measured on sulfite reductase activity and the expression of MET5 and MET10 genes**

|  | **Standardized correlation coefficient (*β*)** | ***P* value** |
| --- | --- | --- |
| **Sulfite reductase activity** |  |  |
| Sulfite (μg.mL^-1^) | -0.71 | 0.348 |
| Selenium (μg.mL^-1^) | 0.51 | 0.477 |
| Coefficient of determination (*R^2^*) | 0.44 |  |
| Residual variable (ᵋ) | 0.74 |  |
| **MET5 expression** |  |  |
| Sulfite ( μg.mL^-1^) | 0.88 | **0.019** |
| Selenium ( μg.mL^-1^) | -0.99 | **0.015** |
| Coefficient of determination (*R^2^*) | 0.98 |  |
| Residual variable (ᵋ) | 0.15 |  |
| **MET10 expression** |  |  |
| Sulfite ( μg.mL^-1^) | 0.88 | 0.044 |
| Selenium ( μg.mL^-1^) | -0.97 | 0.037 |
| Coefficient of determination (*R^2^*) | 0.94 |  |
| Residual variable (ᵋ) | 0.24 |  |

**Table S4. Direct effect (correlation coefficients) of population growth of *S. cerevisiae* on sulfite reductase activity and the expression of MET5 and MET10 genes**

|  | **Standardized correlation coefficient (*β*)** | ***P* value** |
| --- | --- | --- |
| **Sulfite reductase activity** |  |  |
| CFU | -0.84 | 0.047 |
| Coefficient of determination (*R^2^*) | 0.78 |  |
| Residual variable (ᵋ) | 0.46 |  |
| **MET5 expression** |  |  |
| CFU | 0.60 | 0.288 |
| Coefficient of determination (*R^2^*) | 0.36 |  |
| Residual variable (ᵋ) | 0.80 |  |
| **MET10 expression** |  |  |
| CFU | 0.60 | 0.301 |
| Coefficient of determination (*R^2^*) | 0.34 | - |
| Residual variable (ᵋ) | 0.81 | - |

**Table S5. Direct effect (correlation coefficients) of sulfite reductase activity on the expression of MET5 and MET10 genes**

|  | **Standardized correlation coefficient (*β*)** | ***P* value** |
| --- | --- | --- |
| **MET5 expression** |  |  |
| Sulfite reductase activity | -0.53 | 0.363 |
| Coefficient of determination (*R^2^*) | 0.27 |  |
| Residual variable (ᵋ) | 0.85 |  |
| **MET10 expression** |  |  |
| Sulfite reductase activity | -0.52 | 0.367 |
| Coefficient of determination (*R^2^*) | 0.27 |  |
| Residual variable (ᵋ) | 0.85 |  |
